# Short-Term Changes in Occlusal Force Distribution and Masticatory Muscle Activity After Restorative Treatment in Children with Molar–Incisor Hypomineralization: An Exploratory Pilot Study

**DOI:** 10.3390/children13091256

**Published:** 2026-09-16

**Authors:** Afra Nur Savaş, Başak Durmuş, Serdar Gözler, Betül Kargül

**Affiliations:** 1 Department of Pediatric Dentistry, Faculty of Dentistry, Institute of Health Sciences, Marmara University, 34854 Istanbul, Türkiye; 2 Department of Pediatric Dentistry, School of Dentistry, Marmara University, 34854 Istanbul, Türkiye; bkargul@marmara.edu.tr; 3 Department of Prosthodontics, School of Dentistry, Istanbul Atlas University, 34408 Istanbul, Türkiye; serdar.gozler@atlas.edu.tr; 4 School of Dentistry, Queen Mary University of London, London E1 4NS, UK

**Keywords:** molar–incisor hypomineralization, bite force, electromyography, mastication, occlusal force, dentin hypersensitivity, children

## Abstract

**Highlights:**

**What are the main findings?**

- Restoration of symptomatic MIH-affected first permanent molars was followed by a reduction in bilateral occlusal force asymmetry (mean 6.51 pp, 95% CI 0.44–12.57; nominal *p* = 0.041), with all five participants changing in the same direction.
- Dentin hypersensitivity resolved in all five participants, while no statistically detectable changes in the assessed sEMG parameters were observed over the three-month follow-up.

**What are the implications of the main findings?**

- These preliminary, hypothesis-generating findings establish a foundation for adequately powered controlled trials examining functional outcomes after MIH restoration.
- Lateral occlusal force deviation was feasible to measure in this age group and merits evaluation as an exploratory functional outcome in future trials of MIH rehabilitation.

**Abstract:**

**Background/Objectives:** Molar–incisor hypomineralization (MIH) causes post-eruptive enamel breakdown, hypersensitivity, and altered chewing, which may unbalance occlusal force distribution and masticatory muscle activity. The functional consequences of restoring MIH-affected teeth remain poorly understood. This exploratory pilot study assessed the feasibility of measuring short-term changes in occlusal force distribution and masticatory muscle activity after restorative treatment of MIH-affected molars. **Methods:** In this prospective single-arm pilot study, five children (aged 8–10 years) with symptomatic MIH affecting 10 first permanent molars were treated with a bulk-fill glass-hybrid material. Lateral occlusal force distribution (LOFD) was pre-specified as the primary exploratory outcome, bilateral masseter and anterior temporalis surface electromyography during maximum voluntary clenching as a secondary exploratory outcome, and the Schiff Cold Air Sensitivity Scale (SCASS) as an additional exploratory measure. Assessments were performed at baseline and three months. **Results:** All five participants completed follow-up. Mean deviation from bilateral occlusal balance fell from 11.14 ± 6.12 to 4.63 ± 2.89 percentage points (pp); the mean change was 6.51 pp (95% CI 0.44–12.57; *p* = 0.041 nominal; Cohen’s dz = 1.33), and all five children changed in the same direction. Hypersensitivity resolved in all five participants (SCASS = 0 at T1 in every treated tooth); because the 10 teeth were nested within 5 children, this outcome is reported descriptively, with a participant-level sensitivity analysis (*p* = 0.063). No statistically detectable changes in the assessed sEMG parameters were observed over the three-month follow-up. **Conclusions:** In this small exploratory cohort, restoration of MIH-affected molars was followed by resolution of hypersensitivity and a reduction in lateral occlusal force asymmetry, while masticatory muscle activity showed no detectable change. These hypothesis-generating findings should not be read as evidence of efficacy; LOFD was feasible to measure and merits evaluation as an exploratory functional outcome in adequately powered controlled studies. Trial registration: ClinicalTrials.gov NCT07336212.

## 1. Introduction

Molar–incisor hypomineralization (MIH) is a qualitative enamel defect of systemic origin that primarily affects first permanent molars, leading to porous, fragile enamel prone to post-eruptive breakdown (PEB), hypersensitivity, and increased caries risk [1,2]. While the structural consequences and restorative challenges of MIH are well documented, its impact on the functional harmony of the developing stomatognathic system remains comparatively underexplored [2].

When MIH-affected molars are painful, children tend to chew predominantly on the less affected side [3]. Over time, this asymmetric loading pattern can disturb the balance between left and right occlusal forces and alter muscle recruitment, and has been associated with patterns relevant to temporomandibular function [4,5]. Supporting this, a cross-sectional surface electromyography (sEMG) study found that children with unilateral MIH had higher muscle activity in the contralateral masseter and temporalis, suggesting that a localized dental problem can produce measurable neuromuscular changes across the whole system [3].

Despite a well-developed body of work on MIH diagnosis and clinical management, relatively few studies have looked at how this condition affects functional occlusion and muscle coordination [3,6]. Digital assessment tools now make it possible to examine these aspects with greater objectivity. The T-Scan system records occlusal contact timing and relative force distribution, estimating the proportion of total occlusal force carried by each side rather than absolute force in newtons, capturing small left–right asymmetries that articulating paper simply cannot quantify [7]. Surface electromyography complements this by providing a non-invasive measure of masticatory muscle activity and bilateral symmetry during tasks such as maximum voluntary clenching [8]. Together, the two methods offer a more complete picture of how the stomatognathic system is functioning.

Restoring MIH-affected molars aims to eliminate pain, rebuild tooth structure, and re-establish stable occlusal contacts, all of which may support chewing function. Glass-hybrid materials such as Equia Forte HT (GC Corporation, Tokyo, Japan) have been proposed for MIH restorations given their mechanical properties and fluoride-releasing capacity [9,10], although long-term controlled evidence continues to accumulate. Whether pain relief, as measured by tools like the Schiff scale, translates into genuine improvements in occlusal balance and muscle symmetry has not yet been clearly established.

Consequently, this exploratory study used a computerized occlusal analysis system together with sEMG to assess changes in occlusal force distribution and masticatory muscle activity before and three months after restoring MIH-affected molars. Lateral occlusal force distribution was pre-specified as the primary exploratory outcome, sEMG amplitudes and asymmetry indices as secondary exploratory outcomes, and hypersensitivity (SCASS) as an additional exploratory measure; this hierarchy is maintained throughout. Alongside these outcomes, the study addressed explicit feasibility objectives: recruitment yield, protocol adherence, completeness of the functional recordings, and estimation of the variance of the primary outcome to inform the sample size of a future controlled trial. The null hypothesis was that treatment would produce no measurable change in either parameter.

## 2. Materials and Methods

### 2.1. Study Design and Participants

This prospective, single-arm exploratory pilot study used a pre- and post-treatment design. The protocol was approved by the Marmara University Faculty of Medicine Non-Drug and Non-Medical Research Ethics Committee (protocol code 09.2024.806; date of approval 11 October 2024) and was conducted in accordance with the Declaration of Helsinki. The study was prospectively registered at ClinicalTrials.gov (NCT07336212). The study record was first submitted to ClinicalTrials.gov on 2 January 2026, before the recorded study start date of 5 January 2026, and was first publicly posted on 13 January 2026. Written informed consent was obtained from the parents or legal guardians of all participants, and assent was obtained from the children. All procedures were performed at the Department of Pediatric Dentistry, Marmara University Faculty of Dentistry, Istanbul, Turkiye.

Five children (four boys and one girl), aged 8–10 years, were enrolled. A total of 128 children attending the department were screened for MIH and study eligibility; 11 were identified as having MIH and were assessed against the study criteria. Six were excluded because one or more prespecified eligibility criteria were not met (insufficient cooperation with the recording procedures, missing tooth or teeth, or malocclusion). The remaining five consecutive eligible children were enrolled, and no eligible child or family declined participation. To be included, children had to be aged 7–13 years as pre-specified in the protocol and trial registration, have a confirmed MIH diagnosis according to the criteria of Ghanim et al. [11], present with bilateral MIH affecting at least two first permanent molars with post-eruptive enamel breakdown requiring restoration, and report hypersensitivity in the affected molars; bilateral MIH was defined as involvement of at least one first permanent molar on each side of the dentition, irrespective of whether the two affected/restored molars were located in the same or in different dental arches.

Children were excluded if they had a history of orthodontic treatment, craniofacial anomalies, temporomandibular disorders, or parafunctional habits such as bruxism. Additional exclusion criteria were clinically visible facial asymmetry, malocclusions including crossbite or open bite, increased overjet or overbite, any missing permanent teeth; skeletal Class II/III relationship; any systemic condition or medication known to affect neuromuscular function (e.g., muscle relaxants), and inability to cooperate with the T-Scan or sEMG recording procedures. Consequently, none of the included children presented with crossbite, open bite, increased overjet or overbite, or missing teeth; all were in the mixed dentition with an Angle Class I molar relationship and fully erupted first permanent molars at both assessments.

All participants received the same restorative intervention; therefore, random allocation to parallel experimental groups was not applicable in this single-arm pre–post study. As this was a pilot study designed to generate preliminary data and assess feasibility, a formal sample size calculation was not performed. Instead, a convenience sample of five children (providing 10 MIH-affected first permanent molars) was enrolled based on the availability of eligible participants during the recruitment period and the logistical feasibility of advanced functional measurements. No participants were excluded from the final analysis due to missing or incomplete data.

**Figure 1:**
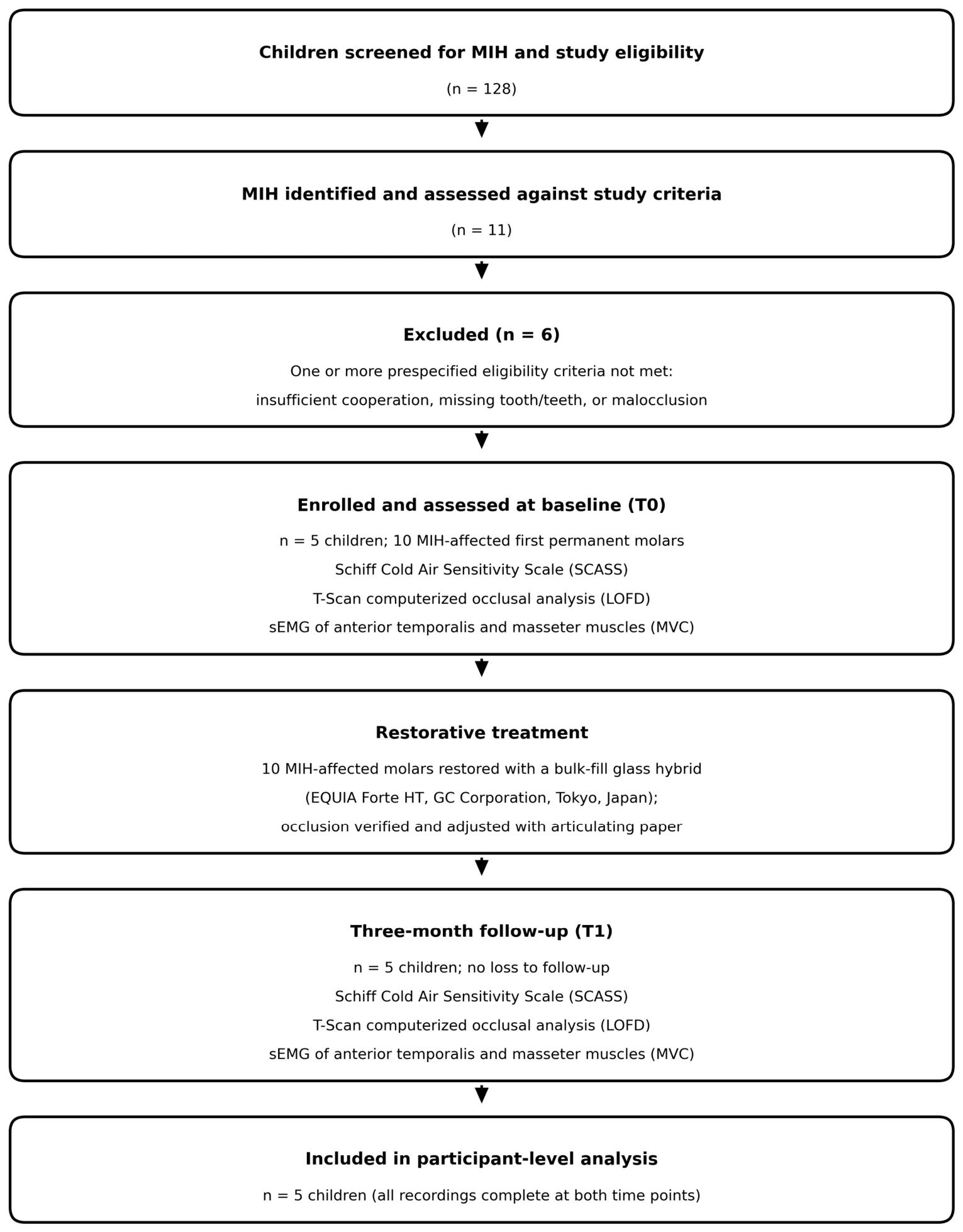
Participant flow through screening, eligibility assessment, enrolment, treatment, three-month follow-up, and analysis, with the assessments performed at each stage. The same three assessments were carried out at baseline and at the three-month follow-up. MIH, molar–incisor hypomineralization; T0, baseline; T1, three-month follow-up; SCASS, Schiff Cold Air Sensitivity Scale; LOFD, lateral occlusal force distribution; sEMG, surface electromyography; MVC, maximum voluntary clenching. All five enrolled children completed follow-up with complete functional recordings at both time points, and no adverse events were recorded.

All participants underwent baseline functional assessments (T0), received standardized restorative treatment, and were re-evaluated three months post-operatively (T1). The first participant was enrolled in January 2026, and all three-month follow-up assessments were completed by May 2026. Participant flow, including the numbers screened, excluded with reasons, enrolled, followed up, and analysed, is summarized in Figure 1.

### 2.2. Outcome Measures and Instrumentation

#### 2.2.1. Lateral Occlusal Force Distribution

Occlusal force distribution was assessed with the T-Scan Novus system running T-Scan 10 software (Tekscan Inc., Boston, MA, USA) (Supplementary Figure S1), a computerized device that captures static and dynamic occlusal contacts, records relative force magnitudes, and tracks contact timing in real time. A small T-Scan^TM^ Novus^TM^ Dental Sensor appropriate for the mixed dentition was used. Sensor sensitivity was calibrated individually for each child at baseline following the manufacturer’s equilibration procedure, and sensitivity settings were reproduced at the three-month visit. During each recording session, the child sat upright with the Frankfort horizontal plane parallel to the floor, and the sensor was placed so that its midline marker aligned with the dental midline. Three recordings were acquired at each session. Repeating the recording served to accustom the child to the sensor and to confirm a correct, fully seated maximum intercuspal bite; it was not a means of selecting among results, and no recording was ever chosen on the basis of the force values it produced. The recording that was technically satisfactory, being free of sensor slippage, incomplete closure and movement artefact, was taken forward for analysis. When more than one recording fulfilled these criteria, the first technically satisfactory recording in acquisition order was retained for analysis; subsequent acceptable recordings did not replace it. No recording was ranked or selected according to the magnitude of the T-Scan force percentage or sEMG amplitude. The same procedure was applied at T0 and T1. Each recording lasted 10 to 15 s and contained a series of five consecutive maximum voluntary clenches in maximum intercuspation (MIP), separated by intervals of approximately one second and delimited automatically by the software from the force–time curve. The first four technically satisfactory clench cycles were used for analysis, while the fifth clench served as a reserve when necessary. Within each clench cycle, the left- and right-side force percentages were extracted at the frame corresponding to peak total force in that cycle; the cycle-specific values were then averaged.

Lateral occlusal force distribution (LOFD) was expressed as the percentage of total occlusal force distributed to the left (FL) and right (FR) sides. In accordance with computerized occlusal analysis reference methodology [7], a 50:50 left–right split was used as the reference anchor for perfect symmetry. Because FL + FR = 100%, the two side-specific percentages are mathematically complementary and |FL − 50| and |FR − 50| are identical; the study therefore has a single bilateral deviation variable rather than two independent asymmetry indices. The primary outcome was this absolute deviation from 50%, expressed in percentage points (pp), with lower values indicating more symmetrical force distribution. The 50:50 anchor is used here as a within-subject reference for change over time and not as a validated physiological target for children in the mixed dentition (see Section 4).

#### 2.2.2. Surface Electromyography

Bilateral sEMG activity of the anterior temporalis (TA) and masseter (MM) muscles was recorded with a BioEMG III device (BioResearch Associates Inc., Milwaukee, WI, USA) interfaced with BioPAK System (version 7.2; BioResearch Associates Inc., Milwaukee, WI, USA) at a sampling rate of 2000 Hz per channel and a fixed acquisition bandwidth of 30–1000 Hz (Supplementary Figure S2). Electrode placement, inter-electrode distance, and signal acquisition followed SENIAM guidelines [12], which provide a standardized framework for sEMG recordings to ensure consistent and reproducible measurements. The skin overlying each muscle was cleaned with 70% alcohol and allowed to dry. Pre-gelled bipolar Ag/AgCl surface electrodes were positioned parallel to the muscle fibre direction with an inter-electrode distance of 20 mm: over the masseter belly along the exocanthion–gonion line, and over the anterior temporalis along the anterior temporal line, both identified by palpation during a light clench. A ground electrode was placed on the skin of the upper back, over the cervical spine serving as an electrically inactive reference site remote from the muscles under study, consistent with the reference-electrode placement options given in the SENIAM recommendations [12]. All electrode placements at both visits were performed by the same examiner using the same anatomical landmarks. All recordings were conducted during maximum voluntary clenching (MVC) in maximum intercuspation under controlled and standardized conditions, consistent with established EMG protocols used in craniofacial and masticatory muscle research [13,14]. sEMG recordings were made simultaneously with the T-Scan recordings during the same MVC task in MIP; the single analysed recording therefore underlies both measurements. Each electromyographic recording lasted approximately 10 s and contained five consecutive maximum voluntary clenches separated by intervals of about 1 to 1.5 s. Five clenches were acquired to ensure that four technically satisfactory clench segments were available for analysis. The acquisition software delimited four technically satisfactory analysis windows and computed the root mean square (RMS) amplitude over the artefact-free steady-state portion of each window, excluding the initial rise and terminal decay. The mean of these four windows yielded a single representative amplitude for each muscle at each time point (Figure S2). The additional fifth clench served as a reserve when four satisfactory windows were available. No recording was selected on the basis of its amplitude. To evaluate muscle balance, asymmetry indices were computed for each muscle pair using the absolute difference between the right and left sides (TA |R − L| and MM |R − L|); elevated values signify increased imbalance between the two sides. Amplitudes are reported as raw µV and were not normalized to a reference contraction; the resulting limitations for between-session comparison, and the dependence of the absolute |R–L| index on overall signal magnitude, are addressed in Section 4.

#### 2.2.3. Hypersensitivity (Exploratory Outcome)

Tooth hypersensitivity was assessed using the Schiff Cold Air Sensitivity Scale (SCASS) [15], which scores a patient’s reaction to a standardized air-blast stimulus. A 1-s air jet from the dental unit triple syringe was directed perpendicular to the buccal surface of each MIH-affected tooth from a distance of approximately 1 cm, at ambient room temperature and with the unit’s standard air pressure setting held constant across visits. Adjacent teeth were isolated with cotton rolls and the operator’s finger so that the stimulus was confined to the test tooth. The child’s response was rated on a scale from 0 (no reaction) to 3 (severe discomfort). The same examiner performed all SCASS assessments at both visits; blinding of the examiner was not feasible in this single-arm design. Since pain on the affected side is a well-known driver of chewing avoidance, SCASS scores were recorded at both baseline and the 3-month follow-up visit.

### 2.3. Restorative Protocol

All restorative procedures were carried out by one calibrated pediatric dentist using material from a single production lot. Teeth were isolated with cotton rolls and a saliva ejector. After selective caries removal, undermined and porous hypomineralized enamel was reduced to firm, sound margins in line with current best-practice guidance [2], and the cavity was conditioned according to the manufacturer’s instructions. The affected teeth were restored with a bulk-fill glass-hybrid system (Equia Forte HT, GC Corporation, Tokyo, Japan) following the manufacturer’s instructions. Each capsule was triturated at 4000 rpm for 10 s and the material was placed in a single increment and shaped. After finishing and polishing, the coat (Equia Forte Coat, GC Corporation) was applied and light-cured for 20 s. Occlusion was then verified with articulating paper and adjusted where needed to eliminate any premature contacts, with adjustment confined to the restored surfaces and performed according to the conventional procedure used for routine posterior restorations. No T-Scan or sEMG recordings were obtained immediately after restoration; the functional assessments reported here were performed only at baseline and at three months. Participants were monitored clinically for adverse events, including pulpal symptoms, restoration loss or fracture, and soft-tissue irritation, at the three-month follow-up visit.

### 2.4. Statistical Analysis

All statistical analyses were carried out using IBM SPSS Statistics version 27 (IBM Corp., Armonk, NY, USA). The unit of analysis is stated separately for each outcome. LOFD deviation and all sEMG variables were analysed at the participant level (*n* = 5, paired comparisons on 4 degrees of freedom). Tooth-specific T-Scan percentages are presented descriptively by tooth position, with the number of contributing participants reported for each position. SCASS was recorded at tooth level (10 teeth) but, because the two treated molars of a child are not independent observations, tooth-level scores are reported descriptively only; the inferential sensitivity analysis uses each participant’s highest score, giving five independent observations. The Shapiro–Wilk test was inspected but was not used as the basis for test selection, because at *n* = 5 it has minimal power and cannot provide a meaningful assessment of normality. With only five paired observations, the distributional assumptions underlying *t*-based analyses could not be assessed reliably. The paired *t*-tests are therefore retained as exploratory *t*-based summaries of mean within-participant change, with the explicit acknowledgement that their validity relies on approximate normality of the paired differences, an assumption that cannot be verified adequately in this dataset. For the primary LOFD outcome, an exact two-sided sign test was additionally reported as a distribution-free sensitivity analysis. Descriptive statistics were reported as mean ± standard deviation (SD) or median with interquartile range (IQR), based on the distribution of each variable. Because FL and FR are complementary, a single inferential test was performed on the bilateral deviation index rather than separate tests on each side. Because SCASS scores are ordinal, pre- and post-treatment values were compared using the Wilcoxon signed-rank test. In line with the exploratory aim, results are reported as paired mean differences with 95% confidence intervals and standardized effect sizes (Cohen’s dz) rather than as significance decisions, and individual participant trajectories are presented graphically for the primary outcome. Given the exploratory nature of this pilot work, no correction for multiple comparisons was applied; all *p*-values are therefore nominal and are interpreted as descriptive summaries of the data rather than as confirmatory tests. Inferential analyses used unrounded values; tabulated values are rounded for presentation.

## 3. Results

### 3.1. Participants

A total of five children (four boys, one girl; mean age ± SD: 9.2 ± 0.8 years) providing 10 MIH-affected first permanent molars were enrolled and completed both the baseline (T0) and 3-month follow-up (T1) assessments. The feasibility objectives were evaluated as follows: of 128 children screened, 11 had MIH and 5 children were enrolled over a one-month window; retention was 100%, every planned T-Scan and sEMG recording was obtained and analysable at both time points, and there were no protocol deviations. No participants were lost to follow-up. No adverse events were recorded in any participant during the study period. The participant flow is summarized in Figure 1. The distribution of MIH severity among the treated teeth, according to the MIH treatment need index (MIH-TNI) [16], was as follows: *n* = 2 teeth scored 4a and *n* = 8 teeth scored 4b. Individual participant characteristics, including sex, age, the FDI positions of the restored molars, tooth-level MIH-TNI scores, and tooth-level SCASS scores at both time points, are given in Table 1. Each child had exactly two MIH-affected first permanent molars restored. Teeth 16 and 26 were each restored in four children, and teeth 36 and 46 in one child each.

### 3.2. Lateral Occlusal Force Distribution (T-Scan)

**Figure 2:**
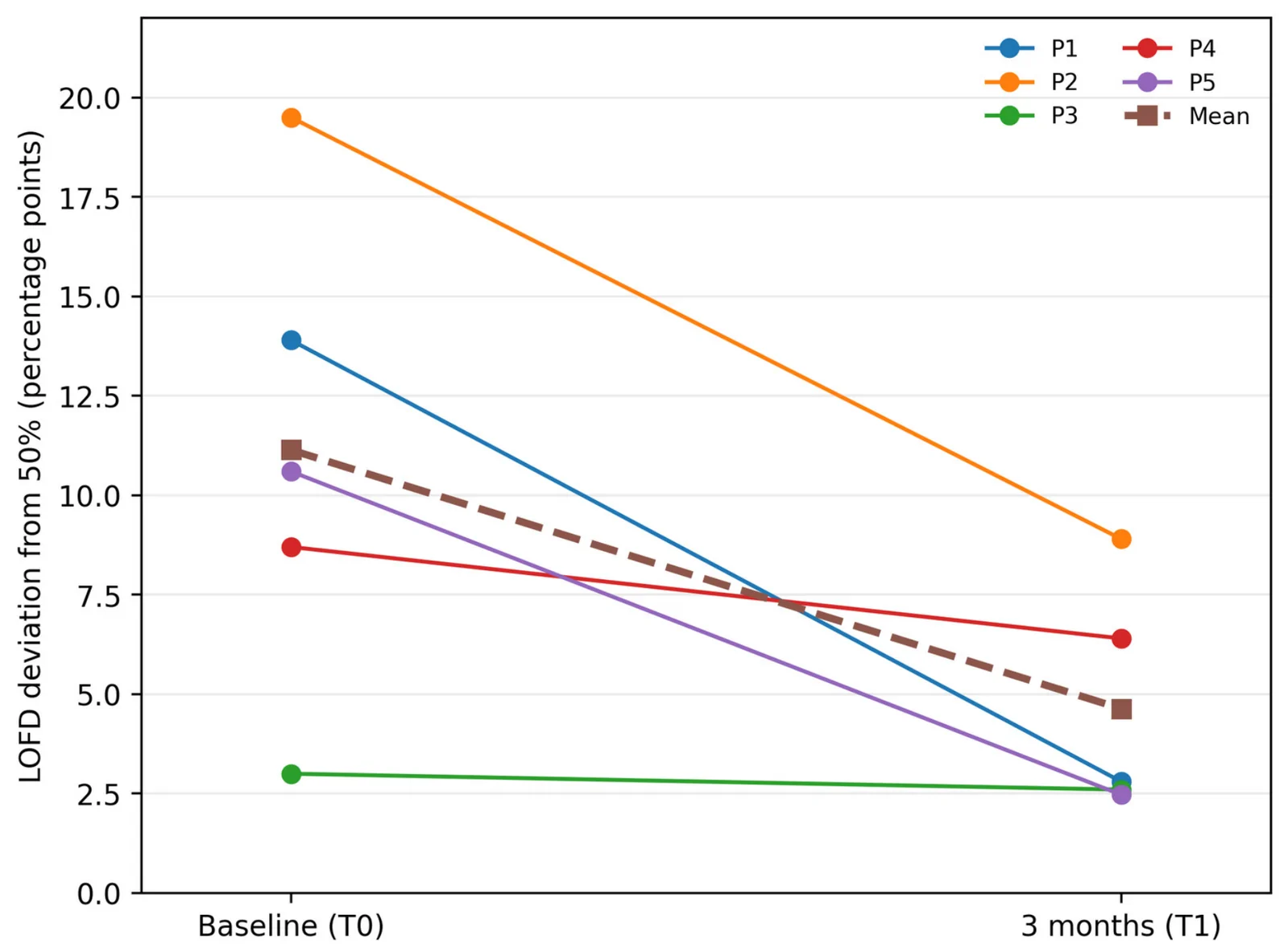
Individual trajectories of lateral occlusal force deviation from bilateral balance between baseline (T0) and the three-month follow-up (T1). Each solid line represents one participant (P1–P5); the dashed line is the group mean. Lower values indicate more symmetrical loading. All five children moved toward bilateral balance, with the largest reductions in the two children who were most asymmetrical at baseline.

Individual LOFD values and their deviations from bilateral balance are given in Table 2, and individual trajectories are plotted in Figure 2. At baseline the direction of asymmetry differed between children: two children loaded predominantly to the left (%R < 50) and three predominantly to the right (%R > 50). All five children showed a reduction in deviation from bilateral balance at three months, although the magnitude varied substantially. As shown in Table 3, the mean deviation from the 50:50 reference for bilateral force balance decreased from 11.14 ± 6.12 pp at baseline (T0) to 4.63 ± 2.89 pp at the three-month follow-up (T1). The mean paired reduction was 6.51 pp (95% CI 0.44 to 12.57; nominal paired *t*-test *p* = 0.041; Cohen’s dz = 1.33). All five participants had lower LOFD deviation at T1; the exact two-sided sign test for this uniform direction of change was *p* = 0.0625. The confidence interval is wide and its lower bound lies close to zero; therefore, these descriptive estimates are imprecise and should not be interpreted as confirmatory evidence of treatment efficacy.

Mean FL and FR values remained close to 50% at both visits and are presented descriptively in Table 3. Because the direction of baseline asymmetry differed between participants and FL and FR are complementary, no separate inferential tests were performed for the side-specific percentages.

Occlusal force was also recorded at each first permanent molar position, but these tooth-level values are not analysed here. The T-Scan percentages are compositional, summing to 100% across the arch, so a change at one position is necessarily offset elsewhere and cannot be read as an isolated gain at that tooth. For transparency, the descriptive values are reported in Supplementary Table S1, without inferential testing, and no conclusion is drawn from them.

### 3.3. Masticatory Muscle Activity (sEMG)

No statistically detectable changes in the assessed sEMG parameters were observed over the three-month follow-up. RMS amplitudes of the anterior temporalis and masseter muscles did not differ significantly between T0 and T1 at any recorded site, as detailed in Table 4 (*p* ≥ 0.05 throughout); the 95% confidence intervals for all paired mean differences include zero. Mean values tended to be slightly lower at follow-up for most muscles, but the wide min–max ranges and large standard deviations at both time points indicate considerable variation between children, which limits what can be read into these shifts.

The asymmetry indices (|TA-R − TA-L| and |MM-R − MM-L|) both moved numerically toward more balanced left–right activity, and the reduction was larger for the masseter than for the temporalis. Neither change reached statistical significance and both confidence intervals span zero. Because these indices are absolute differences in unnormalized µV, they scale with overall signal amplitude, and a numerical reduction is equally compatible with sampling variability, session-to-session measurement differences, or regression toward the mean. They are therefore reported as descriptive observations only.

Taken together, the occlusal and electromyographic findings differed in the present cohort: a change was detectable in occlusal force distribution over three months, whereas none was detectable in the sEMG parameters. Possible reasons for this contrast, and the reasons why it cannot be taken as evidence of a slower neuromuscular response, are considered in the Discussion.

### 3.4. Hypersensitivity (SCASS)

The SCASS scores pointed to a complete resolution of hypersensitivity after treatment. Before restoration, the affected molars produced a clear response to the cold air stimulus, with scores ranging from 1 to 2 (median 1, IQR 1–2). At the 3-month follow-up, every restored tooth scored 0 (median 0, IQR 0–0), meaning none of the children reported any discomfort on retesting. As set out in Section 2.4, the ten treated teeth are nested within five children and are not independent observations, so these tooth-level data are presented descriptively (Table 5) and no tooth-level *p*-value is reported. A participant-level sensitivity analysis, taking each child’s highest SCASS score as the unit of observation (*n* = 5), gave a Wilcoxon signed-rank *p*-value of 0.063. With five participants all changing in the same direction, 0.063 is the smallest value this test can return, so it reflects the size of the sample rather than the strength of the finding. The clinically relevant observation is the uniformity of the finding: every treated tooth in every child reached the lowest possible score, with no residual variation at follow-up.

## 4. Discussion

This exploratory pilot study examined whether restorative rehabilitation of symptomatic MIH-affected molars with a glass-hybrid material is followed by measurable short-term change in functional occlusal parameters. The main observation was a reduction in lateral occlusal force imbalance at three months, with the mean deviation from bilateral balance falling by 6.51 pp (95% CI 0.44 to 12.57; *p* = 0.041 nominal), and all five participants demonstrating a consistent trend toward improved bilateral symmetry (Figure 2). This pattern was not visible in the side-specific force values (FL, FR) themselves, which were essentially unchanged at the group level: because the direction of asymmetry differed between children, left-heavy and right-heavy baselines cancelled out in the group mean even though individual bites were asymmetrical. The deviation index used here is direction-free and therefore retains this information, which is why it—rather than FL or FR individually—is the appropriate outcome for a bilaterally heterogeneous sample. The uniform direction of change across participants is informative in this exploratory dataset, but it cannot establish functional recovery or a treatment effect. Due to the limited sample of five paired observations, the *p*-value remains highly sensitive to individual participant variance, and the resulting wide confidence interval spans a range from a negligible to a large reduction. The finding is best read as a hypothesis-generating signal rather than as evidence of a treatment effect. An alternative explanation that cannot be excluded is regression toward the mean: children were enrolled because they were symptomatic, the two most asymmetrical children at baseline showed the largest reductions, and the child who was already near-symmetrical changed least—a pattern that is also what regression to the mean would produce. This matters clinically because left–right force balance is widely used as a marker of occlusal stability in digital analysis. It should be acknowledged, however, that even clinically healthy dentitions do not always fall within the conventional 50% ± 5% range [17,18]. The available reference values derive largely from adult dentitions, and no validated pediatric thresholds exist for children in the mixed dentition, in whom exfoliation, eruption and occlusal maturation are ongoing. The 50:50 anchor is therefore used here only as a within-subject reference against which each child serves as their own control, and the results should not be described as normalization toward a physiological target.

The baseline imbalance identified in this study aligns with the current literature, as hypersensitivity, structural fragility, and post-eruptive enamel degradation may impair masticatory function on the affected side and encourage protective unilateral loading [6,19]. In our cohort, hypersensitivity resolved completely after restoration, and it is plausible that relief of pain reduced protective avoidance and allowed the affected side to participate more fully in loading. This remains an inference rather than a demonstrated mechanism: habitual chewing side and pain-related avoidance behaviour were not measured, the design contains no mediation analysis, and with five children and a single follow-up point the temporal co-occurrence of symptom relief and occlusal change cannot establish that one produced the other. Persistent unilateral chewing has been associated with altered muscle recruitment and temporomandibular function [4,5]; however, these outcomes were not measured in the present study, and no broader functional benefit can be inferred from the observed LOFD change.

A further explanation for the occlusal finding must be considered explicitly. As part of the standard restorative protocol, occlusion was checked with articulating paper after placement and adjusted to remove premature contacts. That adjustment acts directly on the same domain the primary outcome measures, so the reduction in lateral force deviation cannot be attributed specifically to the resolution of MIH pathology or to any subsequent functional adaptation; it may in part, or even predominantly, reflect the immediate mechanical consequence of restoring tooth contour and equilibrating the occlusion. The present design cannot separate these possibilities, because no T-Scan recording was made immediately after treatment. An immediate post-operative measurement would have partitioned the change into an instantaneous mechanical component and any further change accruing over the following three months, and its absence is a substantive limitation. Future studies of this question should include a recording at the end of the restorative appointment and should document the extent of occlusal adjustment performed.

No sEMG outcome changed detectably over the three months. Although the contrast between the occlusal and electromyographic findings might suggest that neuromuscular adaptation lags behind immediate mechanical changes—as the re-establishment of stable occlusal contacts is immediate, whereas muscle recruitment patterns built up over years of compensatory chewing may reorganize slowly [8,20]—such an interpretation remains a hypothesis for longitudinal work rather than a conclusion supported by the present study. Specifically, two measurements taken three months apart in a sample of five children cannot demonstrate whether adaptation was underway, nor that a longer interval would reveal it. Furthermore, several methodological considerations caution against over-interpreting this null electromyographic finding. Because sEMG amplitudes were recorded as raw values (µV) without normalization to a reference contraction, between-session variations in electrode placement, skin impedance, and subcutaneous tissue thickness could affect the longitudinal comparison independently of any actual physiological change. Under these conditions, the absolute difference index (|R − L|) remains highly dependent on overall signal amplitude rather than serving as a truly normalized measure of symmetry. Additionally, sEMG assessment was restricted to maximum voluntary clenching; dynamic chewing tasks may reveal functional asymmetries or changes in masticatory muscle coordination that are less evident during static clenching [21]. Consequently, subsequent research should integrate functional chewing tasks, normalized amplitude measures, and relative asymmetry indices to improve EMG sensitivity in pediatric populations with MIH.

Tooth-level force values were not analysed, for reasons that are themselves informative about how T-Scan output should be handled. Because all force values are normalized to 100%, a shift toward better bilateral balance tends to be distributed across multiple teeth rather than producing a clear change at any single tooth; the percentages are compositional, so per-tooth values are not independent quantities and an increase at one position is arithmetically inseparable from decreases elsewhere. This is in line with prior work showing that per-tooth force distribution is highly variable between individuals and is not a reliable primary outcome [17]. The tooth-level T-Scan percentages are therefore retained only as descriptive contextual data. Future studies examining tooth-level loading in MIH should use a design in which the status of each analysed tooth position is defined consistently across participants.

The choice of restorative material also bears on the interpretation of these findings and on the design of future work. Glass-hybrid systems are a practical option in this setting because of their moisture tolerance, ease of bulk placement in children with limited cooperation, and fluoride release, although comparative clinical evidence is still accumulating. In this context, recent laboratory investigations have further characterized the fluoride-release kinetics of contemporary pediatric restorative materials [22]. Biocompatibility is a further consideration in this age group, and an in vitro study assessing bisphenol A release from pediatric restorative materials under varied pH and temperature conditions included the glass-hybrid system used here [23]. Neither property was measured in the present study, and neither can be inferred from the functional outcomes reported; they are noted because material selection in MIH involves a balance of handling, longevity, fluoride release and biocompatibility that a functional pilot of this size cannot address, and because a controlled trial comparing restorative materials would need to account for them alongside the occlusal endpoints considered here [2,9,10]. Clinical evidence for glass-hybrid restorations in this age group has also continued to accumulate, including a two-year survival analysis of high-viscosity glass ionomer placed after selective caries removal in MIH-affected molars [24] and a 36-month randomized split-mouth comparison of glass hybrid with short fibre-reinforced composite [25].

From a clinical standpoint, these findings add a functional dimension to MIH research, which has traditionally focused on structural repair and symptom control [2,9]. They also point to a practical question for future trials: whether objective functional measures provide incremental clinical value beyond conventional symptom scores and restoration survival. Although the current pilot study was not designed to address these parameters directly—as it evaluated neither diagnostic accuracy nor predictive capabilities—it did not follow the participants to any clinical endpoint, such as temporomandibular dysfunction, nor did it establish a threshold of change that would count as clinically meaningful. Consequently, no claim can be made that computerized occlusal analysis or surface electromyography (sEMG) can identify pediatric patients at risk of broader stomatognathic problems [19,20].

The developmental stage of the participants deserves particular emphasis. Between 8 and 10 years the dentition and the occlusion are changing continuously through exfoliation, eruption and craniofacial growth, and occlusal contact relationships in the mixed dentition are correspondingly unstable. In the absence of an untreated MIH group or a healthy comparison group followed over the same interval, a three-month change in occlusal force distribution cannot be separated from the change that normal development and ordinary measurement variability would produce in any case. This is an inherent limitation of a single-arm pre–post design in a growing population and is a principal reason why the present findings cannot support causal attribution.

While the preliminary nature of this exploratory pilot study is acknowledged, several methodological limitations warrant a cautious interpretation of the reported findings. The sample of five children was a convenience sample recruited at a single centre, which limits generalizability and leaves the study underpowered for hypothesis testing; the single nominally significant result should be regarded as preliminary. There was no untreated or healthy comparator, no randomization, and no blinding of the examiner performing the assessments, so the observed changes cannot be attributed to the intervention. Occlusal adjustment during restoration acts directly on the primary outcome and constitutes a major potential confounder, compounded by the absence of an immediate post-operative recording. The ten treated teeth are clustered within five children, and tooth-level outcomes are therefore reported descriptively rather than tested. Multiple exploratory comparisons were made across occlusal, electromyographic and sensitivity outcomes without correction, so nominal *p*-values overstate the evidence. All recordings were made during maximum voluntary clenching, so dynamic functions such as chewing efficiency, bolus formation and habitual chewing side were not assessed. Finally, three months is short relative to both neuromuscular adaptation and craniofacial development in this age group. Eruption stage, any MIH-affected teeth beyond the two restored per child, and occlusal characteristics such as Angle relationship, overjet and overbite were recorded and are reported only at the cohort level, not individually for each participant; individual-level detail on these characteristics would strengthen future work of this kind. Despite these constraints, the data provide a reasonable basis for moving toward larger, controlled studies on the functional outcomes of MIH rehabilitation.

Read as a pilot rather than as an efficacy study, the investigation met its feasibility objectives and provides preliminary planning information for future controlled work. Screening 128 children over one month produced 11 with MIH and 5 who met all criteria, indicating that a multi-centre or longer recruitment window will be necessary to reach an adequate sample. Retention was complete and every functional recording was analysable, supporting the practicability of combined T-Scan and sEMG assessment in cooperative children of this age. The observed standard deviation of the within-child change in LOFD deviation was 4.89 pp; on that basis, detecting a 5-pp between-group difference in mean change in LOFD from baseline to follow-up using a two-sample change-score analysis in a two-arm parallel trial with 80% power and a two-sided alpha of 0.05 would yield approximately 16 to 17 participants per group before allowance for attrition. The 5-pp difference was selected as a provisional planning scenario slightly smaller than the 6.51-pp mean within-child change observed in this pilot and is not an established minimal clinically important difference. Because both the variance estimate and the clinically relevant difference remain uncertain, this sample-size estimate should be regarded as illustrative rather than as an established recruitment target. A definitive trial should base its final sample-size calculation on a more reliable variance estimate and include an allowance for attrition. Such a trial should include a comparator group, an immediate post-restoration assessment, normalized electromyography, and dynamic chewing tasks alongside maximum voluntary clenching. Integrated protocols that combine digital occlusal analysis with jaw-tracking and electromyographic recording have been described for the assessment of masticatory and temporomandibular function and offer a practical model for such a design [26].

## 5. Conclusions

In this exploratory pilot study, restoring MIH-affected molars with a glass-hybrid material was followed by complete resolution of hypersensitivity and a reduction in lateral occlusal force deviation at three months, with every child changing in the same direction; no detectable change was observed in masticatory muscle activity. These findings come from five children studied without a control group and should be read as hypothesis-generating, not as evidence that restoration produces functional recovery, nor generalized beyond this cohort. Lateral occlusal force deviation proved feasible to measure and showed a consistent directional change, supporting its evaluation as an exploratory functional outcome; its reliability, responsiveness and minimal clinically meaningful change remain to be established in adequately powered, controlled studies with a comparator group and longer follow-up.

## Figures and Tables

**Table 1:** Individual participant characteristics, restored first permanent molars, MIH-TNI severity scores, and tooth-level SCASS scores before (T0) and after (T1) restorative treatment.

Patient	Sex	Age (y)	Restored FPMs (FDI)	MIH-TNI (Per Tooth)	SCASS T0	SCASS T1
P1	Male	10	16, 36	4a, 4b	2, 1	0, 0
P2	Female	8	16, 26	4a, 4b	2, 2	0, 0
P3	Male	9	26, 46	4b, 4b	1, 2	0, 0
P4	Male	10	16, 26	4b, 4b	1, 1	0, 0
P5	Male	9	16, 26	4b, 4b	1, 1	0, 0

FPM, first permanent molar; FDI, Fédération Dentaire Internationale two-digit notation; MIH-TNI, MIH treatment need index; SCASS, Schiff Cold Air Sensitivity Scale. Each child had exactly two MIH-affected molars restored. Scores are listed in the same order as the corresponding teeth.

**Table 2:** Individual lateral occlusal force distribution before and after restoration, expressed as the right-side share of total occlusal force (%R) and as the absolute deviation from bilateral balance.

Patient	%R at T0	%R at T1	Deviation T0 (pp)	Deviation T1 (pp)	Change (pp)
P1	36.1	52.8	13.9	2.8	−11.1
P2	30.5	41.1	19.5	8.9	−10.6
P3	53.0	52.6	3.0	2.6	−0.4
P4	58.7	43.6	8.7	6.4	−2.3
P5	60.6	47.5	10.6	2.5	−8.1

%R, percentage of total occlusal force carried by the right side; deviation, |%R − 50| in percentage points (pp); lower values indicate more symmetrical loading. Negative change values denote movement toward bilateral balance. Because %L + %R = 100, the deviation is identical whichever side is used as the reference.

**Table 3:** Occlusal force distribution parameters measured by T-Scan before and after restoration. The bilateral deviation index is the primary outcome; FL and FR are complementary and are shown descriptively.

Parameter	Pre-Treatment (T0)		Post-Treatment (T1)		Test Statistic	*p*-Value
	Min–Max	Mean ± SD	Min–Max	Mean ± SD		
Force left (%)	39.4–69.5	52.22 ± 13.66	47.2–58.9	52.47 ± 5.25	-	-
Force right (%)	30.5–60.6	47.78 ± 13.66	41.1–52.8	47.53 ± 5.25	-	-
LOFD deviation (pp)	3.0–19.5	11.14 ± 6.12	2.5–8.9	4.63 ± 2.89	2.978	0.041

FL, force left; FR, force right; LOFD, lateral occlusal force distribution (absolute deviation from the 50:50 reference anchor); SD, standard deviation; Min–Max, minimum–maximum range; pp, percentage points. The deviation index was compared by paired-samples *t*-test: mean change 6.51 pp (95% CI 0.44 to 12.57), t(4) = 2.978, Cohen’s dz = 1.33. Because FL + FR = 100%, the side-specific percentages carry identical deviation information and were not tested separately. Individual values are given in Table 2 and plotted in Figure 2. LOFD deviation: mean paired reduction 6.51 pp (95% CI 0.44 to 12.57); nominal paired *t*-test *p* = 0.041; exact two-sided sign-test *p* = 0.0625. All *p*-values are exploratory and uncorrected for multiplicity.

**Table 4:** Surface electromyography of the anterior temporalis and masseter muscles during maximum voluntary clenching: raw EMG amplitude (µV) before and after MIH restoration.

Variable	Pre-Treatment (T0)		Post-Treatment (T1)		Test Statistic	*p*-Value
	Min–Max	Mean ± SD	Min–Max	Mean ± SD		
TA-R	22.3–78.0	55.50 ± 21.79	11.8–74.5	46.24 ± 27.28	1.955	0.122
TA-L	15.8–103.6	61.02 ± 35.66	24.5–82.5	47.30 ± 24.26	1.561	0.194
MM-R	13.9–39.1	28.84 ± 10.40	16.8–41.7	30.62 ± 11.30	−0.632	0.561
MM-L	16.5–68.4	34.42 ± 21.38	18.6–39.6	27.28 ± 9.04	0.876	0.430
TA |R − L|	6.5–25.6	13.28 ± 7.37	7.3–15.1	12.02 ± 2.86	0.359	0.738
MM |R − L|	1.5–33.2	9.74 ± 13.21	1.9–8.6	4.34 ± 2.74	1.090	0.337

TA-R, right anterior temporalis; TA-L, left anterior temporalis; MM-R, right masseter; MM-L, left masseter; |R − L|, absolute right–left asymmetry index; SD, standard deviation; Min–Max, minimum–maximum range. Paired-samples *t*-tests on 4 degrees of freedom. Paired mean differences (T0 − T1) with 95% confidence intervals: TA-R 9.26 (−3.89 to 22.41); TA-L 13.72 (−10.68 to 38.12); MM-R −1.78 (−9.60 to 6.04); MM-L 7.14 (−15.49 to 29.77); TA |R − L| 1.26 (−8.48 to 11.00); MM |R − L| 5.40 (−8.35 to 19.15). All intervals include zero. Amplitudes are raw µV and were not normalized to a reference contraction; the |R − L| indices are amplitude-dependent and are interpreted descriptively (Section 2.2.2 and Section 4).

**Table 5:** Changes in Schiff Cold Air Sensitivity Scale (SCASS) scores before and after glass-hybrid restorative treatment.

Variable	Pre-Treatment (T0)		Post-Treatment (T1)		Test Statistic	*p*-Value
	Min–Max	Median (IQR)	Min–Max	Median (IQR)		
SCASS score, tooth level (10 teeth in 5 children)	1–2	1 (1–2)	0–0	0 (0–0)	−	Not tested ‡
SCASS score, participant level (highest score per child, *n* = 5)	1–2	2 (1–2)	0–0	0 (0–0)	W = 0	0.063 †

SCASS, Schiff Cold Air Sensitivity Scale; IQR, interquartile range; Min–Max, minimum–maximum range. ‡ Tooth-level values are descriptive only; because the two treated molars of a child are correlated observations, no inferential test was performed at this level. † Participant-level sensitivity analysis using each child’s highest SCASS score as the unit of observation (*n* = 5; Wilcoxon signed-rank test, W = 0, exact two-sided *p*). With five participants all changing in the same direction, 0.063 is the smallest *p*-value this test can return, so it is bounded by the sample size rather than by the size of the change.

## Data Availability

The data presented in this study are available on request from the corresponding author due to privacy restrictions involving pediatric participants.

## Supplementary Materials

The following supporting information can be downloaded at https://www.mdpi.com/article/10.3390/children13091256/s1. Supplementary Figure S1: Representative computerized occlusal analysis recording (T-Scan Novus, Tekscan Inc., Boston, MA, USA) obtained during maximum voluntary clenching in maximum intercuspation, showing the two-dimensional occlusal force map with the left–right force distribution bar, the three-dimensional force view, and the force–time graph. The force–time graph shows the four analyzed clench cycles, delimited by the software’s occlusal event markers (A–D). Five clench cycles were acquired in total; four technically satisfactory cycles were used for analysis, while the fifth served as a reserve when necessary. For each analyzed cycle, the left- and right-side force percentages were extracted at the frame corresponding to peak total force within that cycle and then averaged across the four cycles. The two-dimensional map and the distribution bar correspond to the frame at which the cursor is positioned (8.161 s, 96.09% of the maximum total force registered). The figure illustrates a single participant’s recording and does not represent a group-level result, and all software-generated patient and session identifiers have been removed. The 63.9%/36.1% values displayed on the distribution bar are the left and right shares of total occlusal force in one participant’s baseline recording. Supplementary Table S1: Descriptive tooth-specific occlusal force (%) at each first permanent molar position before and after restoration, with the number of contributing participants and how many of those molars were MIH-affected and restored; values are descriptive only and no inferential tests were performed. Supplementary Figure S2: Representative surface electromyography recording (BioEMG III, BioResearch Associates Inc., Milwaukee, WI, USA) obtained during maximum voluntary clenching, showing raw EMG sweeps, the mean amplitude summary (µV), zoomed traces, and muscle level displays for the right and left anterior temporalis (TA-R, TA-L) and masseter (MM-R, MM-L) muscles. The channels are displayed in this order. The sweep shows the consecutive clenches contained in the recording, separated by intervals of approximately 1 to 1.5 s, and the vertical markers delimit the corresponding analysis windows. The EMG Sweep extends beyond the visible screenshot. Five clenches were acquired, while four technically satisfactory windows were used to calculate the mean RMS amplitude. The EMG Summary panel shows the RMS amplitudes of these four windows and their mean. Amplitudes were computed over the artefact-free steady-state portion of each analysis window, excluding the initial rise and terminal decay. Patient identifiers have been masked.

## Abbreviations

The following abbreviations are used in this manuscript:

MIH	Molar–incisor hypomineralization
PEB	Post-eruptive breakdown
TMD	Temporomandibular disorders
sEMG	Surface electromyography
LOFD	Lateral occlusal force distribution
FL/FR	Force left/force right
TA/MM	Anterior temporalis/masseter
RMS	Root mean square
MVC	Maximum voluntary clenching
MIP	Maximum intercuspation
SCASS	Schiff Cold Air Sensitivity Scale
MIH-TNI	MIH treatment need index
SD	Standard deviation
IQR	Interquartile range
CI	95% confidence interval
FPM	First permanent molar
Pp	Percentage points
