# Supplementary material for: Short-Term Changes in Occlusal Force Distribution and Masticatory Muscle Activity After Restorative Treatment in Children with Molar–Incisor Hypomineralization: An Exploratory Pilot Study
Source: Children (Basel). 2026 Sep 16;13(9):1256. doi: 10.3390/children13091256 (PMC13605308; doi:10.3390/children13091256)
Supplement: Supplementary file 1 [file children-13-01256-s001.zip › Supplementary Table-S1.pdf]

Supplementary Table S1. Descriptive tooth-specific occlusal force (%) at each first permanent molar position before (T0) and after (T1) restorative treatment.

Tooth (FDI)	*n* (R/U)	T0 Min–Max	T0 Mean ± SD	T1 Min–Max	T1 Mean ± SD
16	5 (4/1)	0–20.8	8.50 ± 7.93	0.5–26.7	15.38 ± 13.30
26	5 (4/1)	0–25.2	9.66 ± 10.55	5.9–25.3	12.88 ± 8.52
36	5 (1/4)	0–35.7	18.16 ± 16.05	10–29.6	19.15 ± 6.97
46	5 (1/4)	6.4–23.3	11.94 ± 6.76	16–20.7	17.95 ± 1.79

Tooth positions follow the FDI two-digit notation. T-Scan force values are expressed as the percentage of total occlusal force, normalized to 100% across the arch. n denotes the number of participants contributing a measurement at that position; all five participants contributed one observation at every position, and the descriptive statistics were calculated from these five observations. The values in parentheses give the composition of these five molars as MIH-affected and restored (R) versus unrestored (U). R, restored; U, unrestored; SD, standard deviation; Min–Max, minimum–maximum range.

Note on interpretation. These values are reported for transparency and were not analysed inferentially. Two features preclude formal testing. First, T-Scan percentages are compositional: because the values sum to 100% across the arch, an increase at one tooth position is arithmetically inseparable from decreases elsewhere, so per-tooth percentages are not independent observations and a rise at a restored position does not by itself demonstrate that the tooth has regained functional contact. Second, the rows are contributed by different, partly overlapping subsets of children: teeth 16 and 26 were restored in four participants each, so those rows summarize four restored molars, whereas teeth 36 and 46 were restored in one participant each, so those rows average one restored molar together with four unrestored ones. The rows are therefore not comparable with one another, and no conclusion is drawn from them in the main text.
